# RPL21 interacts with LAMP3 to promote colorectal cancer invasion and metastasis by regulating focal adhesion formation

**DOI:** 10.1186/s11658-023-00443-y

**Published:** 2023-04-16

**Authors:** Jiaxian Zhu, Ting Long, Lingfang Gao, Yan Zhong, Ping Wang, Xiaoyan Wang, Zuguo Li, Zhiyan Hu

**Affiliations:** 1 Department of Pathology, Shenzhen Hospital, Southern Medical University, 1333 Xinhu Road, Shenzhen, 518101 Guangdong People’s Republic of China; 2 Department of Pathology, Nanfang Hospital, Southern Medical University, 1838 Guangzhou Avenue North, Guangzhou, 510515 Guangdong People’s Republic of China; 3 Department of Pathology, School of Basic Medical Sciences, Southern Medical University, Guangzhou, 510515 Guangdong People’s Republic of China; 4 Key Laboratory of Molecular Tumour Pathology of Guangdong Province, Guangzhou, 510515 Guangdong People’s Republic of China

**Keywords:** RPL21, LAMP3, TFEB, Focal adhesion, Colorectal cancer, Migration, Invasion, Metastasis

## Abstract

**Background:**

Metastasis is the leading cause of death among patients with colorectal cancer (CRC). Therefore, it is important to explore the molecular mechanisms of metastasis to develop effective therapeutic targets for CRC. In the present study, ribosomal protein L21 (RPL21) was considered as being involved in promoting CRC metastasis, yet the underlying mechanism requires further investigation.

**Methods:**

Immunohistochemistry, western blotting, and quantitative reverse transcription polymerase chain reaction were performed to measure the expression of RPL21 and lysosome-associated membrane protein 3 (LAMP3) in CRC tissues and cells. Wound healing, transwell migration, and invasion assays were performed to study the migration and invasion of cultured CRC cells. An orthotopic CRC mouse model was developed to investigate the metastatic ability of CRC. Transcriptome sequencing was conducted to identify the genes related to RPL21. The dual-luciferase reporter gene assay was performed to determine the transcriptional activity of transcription factor EB (TFEB). The GST/His pull-down assay was performed to investigate the specific binding sites of RPL21 and LAMP3. The cell adhesion assay was performed to determine the adhesion ability of CRC cells. Immunofluorescence staining was performed to observe focal adhesions (FAs).

**Results:**

RPL21 was highly expressed in CRC, contributing to tumor invasiveness and poor patient prognosis. Functionally, RPL21 promoted the migration and invasion of CRC cells in vitro and tumor metastasis in vivo. Moreover, LAMP3 was identified as being highly related to RPL21 and was essential in promoting the migration and invasion of CRC cells. Mechanistically, RPL21 activated the transcriptional function of TFEB to upregulate LAMP3 expression. RPL21 directly bound to the aa 341–416 domain of LAMP3 via its aa 1–40 and aa 111–160 segments. The combination of RPL21 and LAMP3 enhanced the stability of the RPL21 protein by suppressing the degradation of the ubiquitin–proteasome system. Furthermore, RPL21 and LAMP3 promoted the formation of immature FAs by activating the FAK/paxillin/ERK signaling pathway.

**Conclusions:**

RPL21 promoted invasion and metastasis by regulating FA formation in a LAMP3-dependent manner during CRC progression. The interaction between RPL21 and LAMP3 may function as a potential therapeutic target against CRC.

**Supplementary Information:**

The online version contains supplementary material available at 10.1186/s11658-023-00443-y.

## Background

At present, colorectal cancer (CRC) is the third most common cancer diagnosed worldwide, accounting for 8% of all incident cases [1]. Although there have been considerable advances in the early detection, surgical techniques, and targeted therapies against cancer in the past decade, CRC remains the second leading cause of cancer-related deaths. Tumor metastasis, and deleterious liver and lung metastases in particular, decreases the median overall survival of patients with CRC to 30 months [2]. Therefore, exploring the molecular mechanisms of tumor metastasis could unravel potential targets for intervention, which is particularly important for developing effective therapeutics for CRC.

The ribosome is an 80S cellular translational machine comprising a small (40S) and a large (60S) subunit. It is a complex of four ribosomal RNAs (rRNAs) and approximately 80 ribosomal proteins (RPs). The primary function of the ribosome is protein synthesis [3, 4]. Although RPs have long been thought to be only involved in protein translation, recent studies have reported that they can mediate some cellular functions independent of the ribosome machinery, including DNA repair, cell proliferation and differentiation, development, immune signaling, and tumorigenesis [5, 6]. At present, the altered expression and dysfunction of RPs in cancer are the focus of attention as they function either as oncoproteins or tumor suppressors [7]. However, the mechanisms underlying the various functions of RPs in tumorigenesis need further investigation.

The human *RPL21* gene is located on chromosome 13q12.2. It encodes the 60S subunit ribosomal protein L21, which contains 160 amino acids and belongs to the ribosomal protein L21e family. RPL21 is a highly conserved protein in various species, including simple eukaryotes, invertebrates and vertebrates. *RPL21* is the key regulatory gene in embryogenesis and odontogenesis [8]. Mutations in this gene result in human age-related cataracts and hereditary hypotrichosis simplex (HHS) [9, 10]. Nevertheless, there are few studies on the role of RPL21 in tumorigenesis. Studies have reported that RPL21 may act as a biomarker for cervical intraepithelial neoplasia (CIN1) [11]. The siRNA against the *RPL21* gene inhibits pancreatic cancer cell proliferation and DNA replication and induces cell cycle arrest in the G1 phase and apoptosis [12]. Moreover, RPL21 is markedly upregulated in metastatic 5-8F compared with nonmetastatic 6–10B nasopharyngeal carcinoma cell lines, which is associated with tumor metastasis [13]. These studies suggest that RPL21 can contribute to the development of various cancers. However, the potential role of RPL21 in CRC progression remains unclear.

Lysosome-associated membrane proteins (LAMPs) are a family of membrane-bound glycosylated proteins in lysosomes and comprise five members (LAMP1, LAMP2, LAMP3/DC-LAMP, LAMP4, and LAMP5). All these proteins contain evolutionarily conserved LAMP domains [14]. LAMP3/DC-LAMP is a highly glycosylated single transmembrane protein that is expressed in specific cell types and conditions. Studies have reported that LAMP3 is overexpressed in multiple cancer types and that it is associated with tumor metastasis and poor patient prognosis [15–18]. Overexpression of LAMP3 in the human cervical cell line TCS and breast cancer cells promoted cell migration in vitro and lymph node metastasis in vivo [19, 20]. Further, loss of LAMP3 markedly inhibited the motility and metastasis of esophageal cancer cells by activating local PKA expression and promoting VASP phosphorylation at Ser239 [21]. At present, studies on the role of LAMP3 in CRC are limited to its upregulation in samples. Therefore, studies are required to further understand the role of LAMP3 in CRC metastasis.

In the present study, we demonstrated that the upregulation of RPL21 promotes the migration and invasion of CRC cells. For the first time, we report on the interaction between RPL21 and LAMP3, which regulates the formation of focal adhesions (FAs). Our findings offer molecular insights into the prometastatic effect of RPL21 and LAMP3, and suggest their role as therapeutic targets against CRC metastasis.

## Materials and methods

### Antibodies, small-interfering RNA (siRNA), and primer sequences

The antibodies used in this study are listed in Additional file 1: Table S1. The primer sequences used in this study are listed in Additional file 2: Table S2. The siRNA sequences used in this study are listed in Additional file 3: Table S3.

### Cell lines and cell culture

Human normal colorectal epithelial cell line FHC, human CRC cell lines HCT15, Caco2, HCT116, Lovo, SW620, HT-29, SW480, DLD1, RKO, and HCT-8; human embryonic kidney 293T cell line HEK293T were obtained from the American Type Culture Collection (ATCC). All human CRC cells were cultured in RPMI-1640 medium (Gibco, Massachusetts, USA) supplemented with 10% fetal bovine serum (FBS) (ExCell Bio, Guangzhou, Guangdong, China). FHC cells were cultured in RPMI-1640 medium supplemented with 20% FBS. HEK293T cells were cultured in Dulbecco’s modified Eagle medium (DMEM) (Gibco) supplemented with 10% FBS. All cells were cultured at 37 °C in a humidified atmosphere of 5% CO_2_.

### Plasmid construction

Dual-luciferase reporter gene plasmids constructed with pmirGLO vector, *TFEB*-Flag, *RPL21*-GST, *LAMP3*-6His full length plasmids, and their truncations indicated in Fig. 5C constructed with pcDNA3.1(+) vector were purchased from RuiBiotech (Guangzhou, Guangdong, China). *RPL21*-GST and *LAMP3*-6His plasmids constructed with CV129 vector were purchased from Genechem (Shanghai, China).

### Construction of stable cell lines

The lentivirus vector CV146 containing human *RPL21* full-length overexpression sequence (*RPL21*) and the lentivirus vector GV344 containing the indicated *RPL21*-repressing short hairpin RNA sequence (GCACUCUAAGAGCCGAGAU) (*shRPL21*) with empty vectors CV146 and GV344 were constructed by Genechem. The stable cell lines were established as described by the manufacturer.

### Identification and quantification of tumor budding

According to the International Tumor Budding Consensus Conference (ITBCC) 2016 [22], a single tumor cell or a cell cluster consisting of four tumor cells or less was identified as tumor budding. Tumor budding was counted on hematoxylin–eosin (H&E) and assessed in one hotspot (in a field measuring 0.785 mm^2^) at the invasive front. The three-tier system was used as follows: 0–4 buds—low budding (Bd 1), 5–9 buds—intermediate budding (Bd 2), ≥ 10 buds—high budding (Bd 3).

### Immunohistochemistry (IHC)

Paraffin-embedded human CRC dissected tissues were baked in an oven at 70 °C for 2 h. The sections were then dewaxed in the xylene solution, rehydrated in a decreasing concentration of ethanol, and rinsed under running water. The antigens were unmasked in a pressure cooker with the citrate repair solution (ZSGB-bio, Beijing, China) at 2100 W for 30 s, followed by adjusting to 800 W for 10 min and cooling to room temperature. The endogenous peroxidases were blocked by the 3% hydrogen peroxide solution for 15 min away from light. The sections were then incubated with primary antibodies at 4 °C overnight and incubated with a goat anti-rabbit secondary antibody (ZSGB-bio) for 30 min at 37 °C. DAB solution (ZSGB-bio) was used for color development. After hematoxylin staining, the sections were dehydrated in an increasing gradient of ethanol and xylene. Two experienced pathologists independently observed and scored the degree of staining in the sections. The staining intensity was scored as follows: 0 = no staining, 1 = light yellow, 2 = brownish yellow, and 3 = brown.

### Western blotting

Total protein from cells or tissues was lysed by radioimmunoprecipitation assay (RIPA) buffer with with phenylmethylsulfonyl fluoride (PMSF), protease inhibitors, and phosphatase inhibitors (FDbio, Hangzhou, Zhejiang, China). The protein was separated by sodium dodecyl-sulfate polyacrylamide gel electrophoresis (SDS–PAGE) and transferred onto polyvinylidene fluoride (PVDF) membranes. The membranes were blocked by 5% skim milk (FDbio) or 5% BSA (FDbio) for 1 h at room temperature and incubated with primary antibodies at 4 °C overnight. The membranes were then incubated with the corresponding secondary antibodies (FDbio) for 1 h at room temperature. The protein was detected by ECL chemiluminescence solution (FDbio) and visualized by using the chemiluminescence detection system (Bio-Rad, California, USA).

### Quantitative reverse transcription polymerase chain reaction (qRT–PCR)

Total mRNA of CRC cells was extracted with TRIzol reagent (TaKaRa, Beijing, China). The mRNA was reverse transcribed into cDNA by using the Prime-Script RT Reagent Kit (TaKaRa) under the following reaction conditions: 37 °C for 15 min, 85 °C for 5 s, and 4 °C hold. Then, qRT–PCR analysis was performed by using the SYBR Premix Ex Taq (TaKaRa) and the Applied Bio-systems 7500 Fast Real-Time PCR System (Thermo Fisher, Massachusetts, USA). The program was set as preheat at 95 °C for 2 min, 40 cycles of denaturation at 95 °C for 10 s, annealing at 61 °C for 34 s, and a final extension at 72 °C for 30 s, then at 95 °C for 1 min, at 55 °C for 1 min, at 95 °C for 15 s, and 60 °C for 15 s. The quantification of the mRNA level was normalized to GAPDH and calculated by the 2^−ΔΔCt^ method.

### Immunofluorescence (IF) staining

IF staining was performed as described previously [23]. The images were acquired by using a fluorescence microscope (Leica, Germany).

### Wound healing assay

The cells were seeded in a six-well plate and cultured to 90% confluence. The wounds were scratched with a sterile 10 μL pipette tip on a cell monolayer. The cells were cultured in a serum-free medium for 48 h. The images were acquired using an ordinary optics microscope (Olympus, Japan) at 0 h and 48 h. The wound-healing percentage was calculated according to this formula: (the size of the wound at 0 h − the size of the wound at 48 h)/the size of the wound at 0 h.

### Transwell migration and invasion assays

The transwell migration and invasion assays were performed as described previously [23]. The images were acquired by using an ordinary optics microscope (Olympus).

### Cell adhesion assay

A 24-well plate was coated with fibronectin (BD Biosciences, California, USA) at 37 °C for 1 h, followed by blocking with 1% FBS for 30 min. Then, 3 × 10^4^ cells were seeded in each well and incubated at 37 °C for 1 h, rinsed in phosphate-buffered saline (PBS) buffer, fixed with formalin, stained with hematoxylin, and enumerated by an ordinary optics microscope (Olympus).

### Orthotopic injection CRC mouse model

Four 6-week-old male BALB/C-nude mice were purchased from GemPharmatech (Nanjing, Jiangsu, China) and housed under pathogen-free conditions. HCT116 control (Ctrl) or HCT116 RPL21 cells were suspended in the RPMI-1640 medium up to a concentration of 2 × 10^7^ cells/100 μL and then injected into the cecal serosal layers of nude mice. After 8 weeks, the mice were intraperitoneally administered 15 mg/mL of d-luciferin potassium salts (Promega, Beijing, China) at a dose of 10 μL/g. The tumor progression in mice was detected by the IVIS Spectrum In Vivo Imaging System (PerkinElmer, Massachusetts, USA). Then, the orthotopic tumor and livers were surgically removed, imaged, fixed in 10% formalin, embedded in paraffin and sliced into 2.5 μm thick sections for H&E staining.

### Dual-luciferase reporter gene assay

The experimental cells were seeded in a six-well plate and transfected with dual-luciferase reporter gene plasmids. After 48 h, activity of firefly luciferase and Renilla luciferase was detected as per the instruction of the Dual-Luciferase Reporter Assay Kit (TransGene, Beijing, China). The firefly luciferase activity was normalized to the Renilla luciferase activity.

### Co-immunoprecipitation (Co-IP)

Total protein from the cells was lysed by RIPA buffer with PMSF, protease inhibitors, and phosphatase inhibitors (FDbio), followed by subjecting to IP with primary antibodies against RPL21 or LAMP3 in a Protein A/G agarose IP Reagent (Bioworld, Minnesota, USA) at 4 °C overnight. The input was used as a positive control and normal rabbit immunoglobulin (Ig)G was used as a negative control. The immunoprecipitated protein was detected by western blotting.

### GST/His pull-down assay

BL21 (DE3) (TransGene) was transfected with *Ctrl*-GST, *RPL21*-GST, and *LAMP3*-6His prokaryotic plasmids and cultured in the Luria–Bertani (LB) with ampicillin. The corresponding fusion protein was induced by 0.5 mM isopropyl-β-d-1-thiogalactopyranoside (IPTG) (TransGene) at 16 °C for 16 h and harvested by GST-tag or His-tag magnetic beads (Beaver Biosciences, Guangzhou, Guangdong, China). The purified Ctrl-GST and RPL21-GST protein was eluted from the magnetic beads and combined with LAMP3-6His at 4 °C overnight. The remaining protein complex combined with His-tag magnetic beads was detected by Coomassie brilliant blue staining and western blotting. HEK293T cells were cultured and transfected with *RPL21*-GST/*LAMP3*-6His full length or/and truncation plasmids. After 48 h, the target protein was lysed in the RIPA buffer (FDbio) and captured by GST-tag or His-tag magnetic beads. The protein complex combined with GST-tag or His-tag magnetic beads was detected by western blotting.

### Statistical analysis

SPSS 24.0 was used to perform the statistical analyses. An unpaired two-tailed Student’s *t*-test was applied to analyze the differences between two groups of normally distributed data. A paired *t-*test was applied to analyze the difference between the expression of RPL21/LAMP3 in fresh human CRC tissues and paired adjacent normal tissues. Pearson’s chi-squared test was performed to analyze the correlation between RPL21 expression position and clinicopathological features. Kendall’s Tau test was applied to analyze the correlation between RPL21 or LAMP3 expression in tumor buds and the grades of tumor budding, and the correlation between RPL21 and LAMP3 expression in the tumor buds. Kaplan–Meier CRC disease-free survival curves were plotted and a log-rank test was performed. Univariate and multivariate Cox proportional hazards models were used to analyze the correlations between individual parameters and CRC relapse-free survival rate. All data were presented as the mean ± standard deviation. *p* ≤ 0.05 was considered to indicate statistical significance.

## Results

### RPL21 is upregulated in CRC tissues, correlating with tumor invasiveness and patient survival rate

**Fig. 1:**
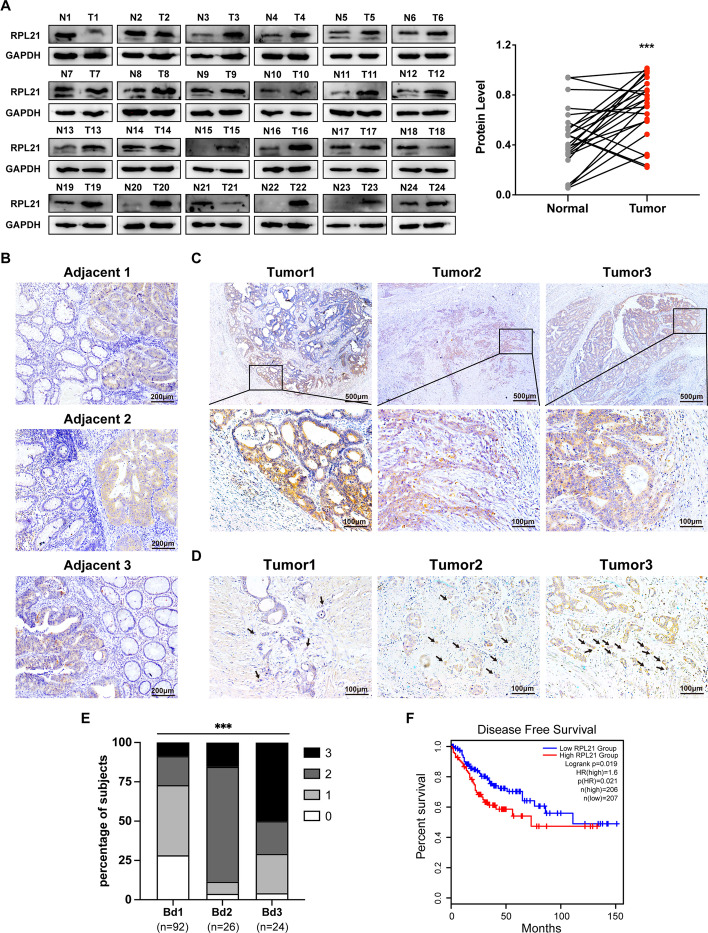
RPL21 is upregulated in CRC tissues, correlating with tumor invasiveness and patient survival rate. **A** RPL21 protein expression in human CRC tissues (T) and paired adjacent normal tissues (N) was detected by western blotting. The quantification of the protein level was normalized to that of GAPDH. *n* = 24, ***p < 0.001, paired Student’s *t*-test. **B** The representative images of the RPL21 expression in 142 paraffin-embedded human CRC tissues with adjacent normal colorectal tissues (adjacent 1, adjacent 2, and adjacent 3) detected by IHC staining (scale, 200 μm). **C** The representative images of RPL21 expression in the invasive front of CRC (tumor 1, tumor 2, and tumor 3) were detected by IHC staining (scale, 500 μm, 100 μm). **D** The representative images of RPL21 expression in CRC tumor buds (tumor 1, tumor 2, and tumor 3) were detected by IHC staining (scale, 100 μm). The arrowheads indicate the tumor buds. **E** The correlation between the scores of RPL21 expression in tumor buds and the grades of tumor budding. *n* = 142, ***p < 0.001, Kendall’s Tau test. **F** Kaplan–Meier survival analysis of CRC patients with high or low expression of RPL21. *n* = 413

To explore the possible role of RPL21 in tumor progression, we investigated the RPL21 expression in various cancers from the Gene Set Cancer Analysis platform GSCA (Additional file 4: Table S4) and observed a remarkable increase in its expression in CRC samples (Additional file 5: Fig. S1A). Then, we investigated the transcriptional level of *RPL21* using the Gene Expression Omnibus databases GSE83889 and GSE87211, and observed higher RPL21 expression in CRC tumor samples than in normal counterparts (Additional file 5: Fig. S1B, C). To confirm this finding, western blotting was performed to detect the protein expression of RPL21 in 24 fresh human CRC tissues (T) and paired adjacent normal tissues (N). Significantly higher RPL21 expression was observed in CRC tissues than in adjacent normal tissues (Fig. 1A).

Next, we performed IHC staining of 142 paraffin-embedded CRC sections to observe the RPL21 expression in situ and found that RPL21 was mainly expressed in CRC tissues rather than in normal colon tissues (Fig. 1B). Notably, RPL21 had higher expression in the invasive front than in the central tumor area (Fig. 1C). Then, we analyzed the correlation between RPL21 localization and the clinicopathological characteristics of CRC. We observed that RPL21 expression in the invasive front was positively correlated with invasion depth and the risk of distant metastasis (Table 1). Moreover, we analyzed the correlation between RPL21 expression and distant metastasis of CRC using the TCGA-COAD database and confirmed that high RPL21 expression in CRC tissues probably results in distant metastasis (Additional file 5: Fig. S1D). Therefore, we focused on RPL21 expression in tumor buds, an independent predictor of CRC metastasis [22], and observed that high RPL21 expression in tumor buds was positively correlated with high-grade tumor budding (Fig. 1D, E).

**Table 1:** The correlation between RPL21 expression and CRC clinicopathological characteristics

	No. of cases	Highly expressed in invasive front?		Chi-squared value	*p*-Value
		Yes	No		
Frequency	142 (100.0%)	95 (66.9%)	47 (33.1%)		
Age					
Younger (< 50 years)	32 (22.5%)	20 (62.5%)	12 (37.5%)	0.361	0.548
Older (≥ 50 years)	110 (77.5%)	75 (68.2%)	35 (31.8%)		
Gender					
Male	87 (61.3%)	60 (69.0%)	27 (31.0%)	0.432	0.511
Female	55 (38.7%)	35 (63.6%)	20 (36.4%)		
Position					
Colon	102 (71.8%)	70 (68.6%)	32 (31.4%)	0.487	0.485
Rectum	40 (28.2%)	25 (62.5%)	15 (37.5%)		
Tumor size (maximum diameter)					
< 5 cm	70 (49.3%)	45 (64.3%)	25 (35.7%)	0.427	0.514
≥ 5 cm	72 (50.7%)	50 (69.4%)	22 (30.6%)		
Tumor grade (differentiation)					
G1	12 (8.5%)	6 (50.0%)	6 (50.0%)	6.221	0.045
G2	106 (74.6%)	77 (72.6%)	29 (27.4%)		
G3	24 (16.9%)	12 (50.0%)	12 (50.0%)		
Invasive depth					
Submucosal	3 (2.1%)	0 (0.0%)	3 (100.0%)	9.457	0.024
Myometrium	16 (11.3%)	8 (50.0%)	8 (50.0%)		
Subserosal	90 (63.4%)	62 (68.9%)	28 (31.1%)		
Break the serosa	33 (23.2%)	25 (75.8%)	8 (24.2%)		
Mucinous component					
Absent	127 (89.4%)	88 (69.3%)	39 (30.7%)	3.101	0.078
Present	15 (10.6%)	7 (46.7%)	8 (53.3%)		
Microsatellite instability					
MSS	119 (83.8%)	81 (68.1%)	38 (31.9%)	1.105	0.575
MSI-L	8 (5.6%)	4 (50.0%)	4 (50.0%)		
MSL-H	15 (10.6%)	10 (66.7%)	5 (33.3%)		
Lymphatic metastasis					
Negative	73 (51.4%)	47 (64.4%)	26 (35.6%)	0.430	0.512
Positive	69 (48.6%)	48 (69.6%)	21 (30.4%)		
Distant metastasis					
Negative	133 (93.7%)	86 (64.7%)	47 (35.3%)	4.754	0.029
Positive	9 (6.3%)	9 (100.0%)	0 (0.0%)		

Kaplan–Meier survival analysis from GEPIA2 (Additional File 4: Table S4) revealed that patients with a higher RPL21 expression had poorer CRC prognosis (Fig. 1F). Further, univariate and multivariate Cox proportional hazards models revealed that RPL21 expression was an independent prognostic factor for patients with CRC (Additional file 6: Table S5, database: GSE39582). Taken together, our results suggest that the upregulation of RPL21 in CRC tissues contributes to tumor invasiveness and poor patient prognosis.

### RPL21 promotes CRC cell migration, invasion, and liver metastasis

**Fig. 2:**
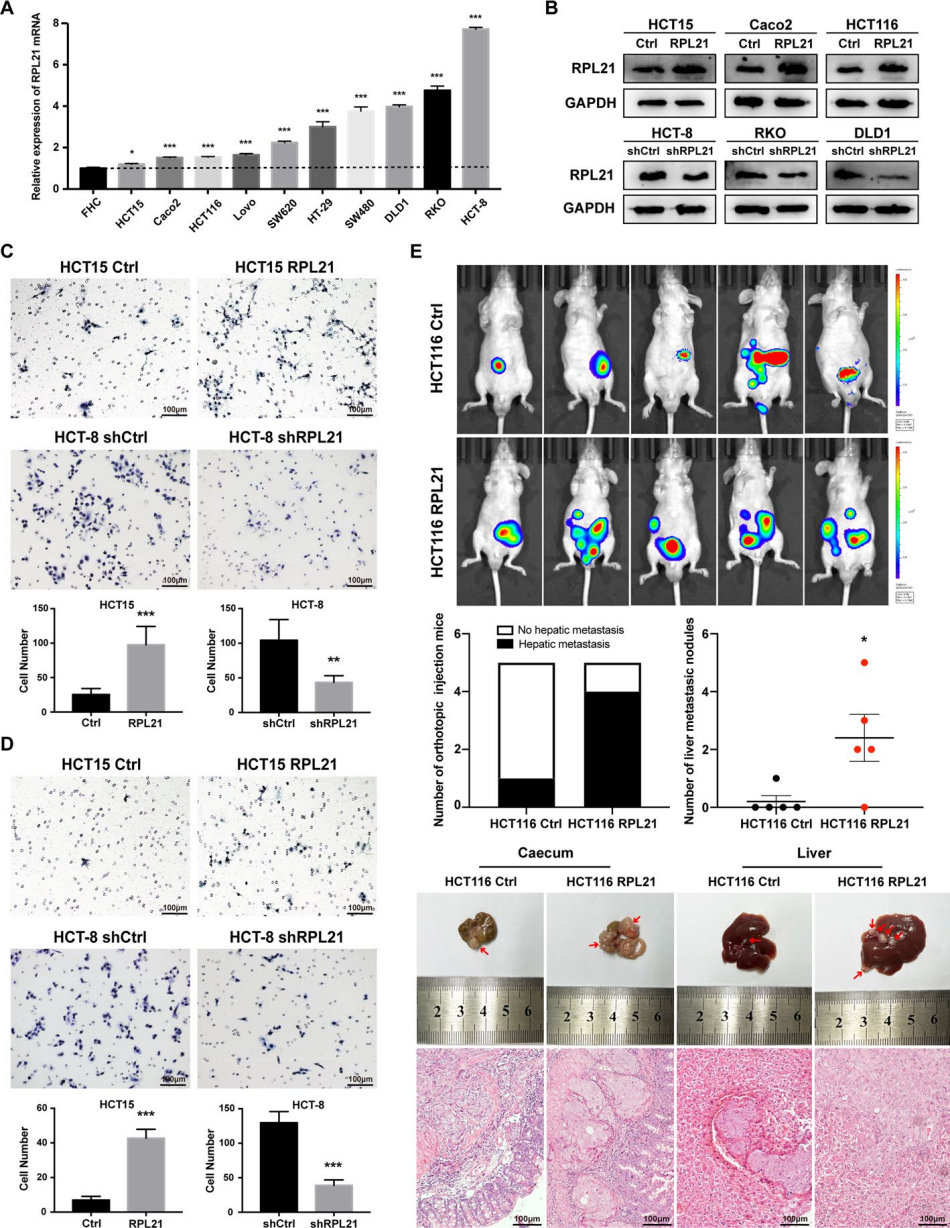
RPL21 promotes CRC cell migration, invasion and liver metastasis. **A** The relative expression of RPL21 mRNA in ten CRC cell lines and FHC was detected by qRT–PCR. Mean ± SD, *n* = 3, ***p < 0.001, *p < 0.05, Student’s *t*-test. **B** The establishment of RPL21 stable overexpression (HCT15, Caco2, and HCT116) and knockdown (HCT-8, RKO, and DLD1) cell lines. RPL21 protein expression was detected by western blotting. **C** The migration ability of CRC cells was detected by the transwell migration assay (scale, 100 μm). Mean ± SD, *n* = 5, ***p < 0.001, **p < 0.01, Student’s *t*-test. **D** The invasion ability of CRC cells was detected by Matrigel-coated Boyden chamber invasion assay (scale, 100 μm). Mean ± SD, *n* = 5, ***p < 0.001, Student’s *t*-test. **E** In vivo bioluminescence imaging photo and representative morphology images with H&E staining images of tumor formation in mouse cecum and liver metastasis (scale, 100 μm), using the orthotopic CRC mouse model injected with HCT116 Ctrl or HCT116 RPL21 cells. The red arrowheads indicate the tumors in situ or the liver metastatic nodules. The number of liver metastatic nodules in each mouse was counted under the microscope. Mean ± SD, *n* = 5, *p < 0.05, Student’s *t*-test

To further investigate the effect of RPL21 on CRC cells, we examined the mRNA and protein expression of RPL21 in ten CRC cell lines and the normal colorectal epithelial cell line FHC. We found that RPL21 expression was higher in all ten CRC cell lines than in the FHC cell line (Fig. 2A and Additional file 7: Fig. S2A). IF staining revealed the localization of RPL21 (green) in CRC cells with ribosomal marker RPS6 (red) as a reference. We found that there was a large part of RPL21 accumulated in the cytoplasm in a ribosome-free form (Additional file 7: Fig. S2B). Then, we established the RPL21-overexpressing HCT15, Caco2, and HCT116 cell lines and RPL21-knockdown HCT-8, RKO, and DLD1 cell lines (Fig. 2B). The wound healing assay and transwell migration and invasion assays revealed that upregulation of RPL21 enhanced the migration and invasion of CRC cells and that its downregulation had the opposite effect (Fig. 2C and D and Additional file 7: Fig. S2C–E).

The effect of RPL21 expression on CRC tumor metastasis in vivo was assessed by preparing a CRC liver metastasis model. Eight weeks after injecting HCT116 Ctrl/RPL21 cells into the cecal serosal layers in nude mice, tumors were formed in situ. Four of the five mice in the HCT116 RPL21 group exhibited liver metastasis. The maximum number of metastatic nodules was five. In contrast, only one mouse in the HCT116 Ctrl group exhibited liver metastasis with one metastatic nodule (Fig. 2E). These data suggest the role of RPL21 in CRC cell migration and invasion in vitro and tumor metastasis in vivo.

### LAMP3 is significantly correlated with RPL21 and promotes the migration and invasion of CRC cells

**Fig. 3:**
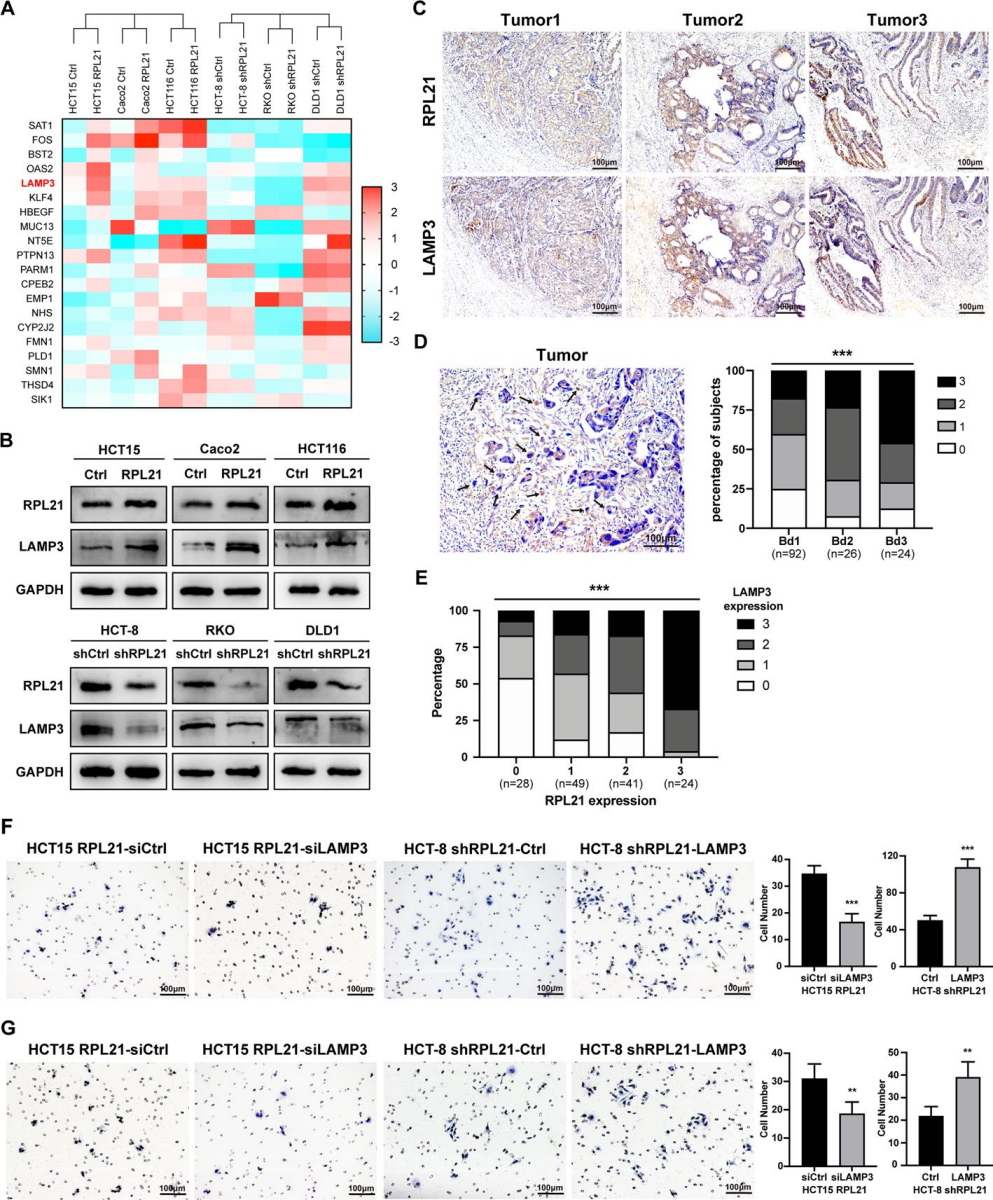
LAMP3 is significantly correlated with RPL21 and promotes the migration and invasion of CRC. **A** Heatmap of the top 20 differentially expressed genes in the transcriptome sequencing of RPL21 stable overexpression and knockdown cells with their control cells. **B** LAMP3 protein expression in RPL21 stable overexpression and knockdown cells detected by western blotting. **C** The representative images of RPL21 and LAMP3 expression in 142 paraffin-embedded human CRC tissues (tumor 1, tumor 2, and tumor 3) detected by IHC staining (scale, 100 μm). **D** The representative image of LAMP3 expression in CRC tumor buds was detected by IHC staining (scale, 100 μm). The arrowheads indicate the tumor buds. The correlation between the scores of LAMP3 expression in tumor buds and the grades of tumor budding was analyzed. *n* = 142, ***p < 0.001, Kendall’s Tau test. **E** The correlation between the scores of RPL21 and LAMP3 expression in tumor buds was analyzed. *n* = 142, ***p < 0.001, Kendall’s Tau test. **F** The migration ability of the indicated CRC cells was detected by the transwell migration assay (scale, 100 μm). Mean ± SD, *n* = 5, ***p < 0.001, Student’s *t*-test. **G** The invasion ability of indicated CRC cells was detected by Matrigel-coated Boyden chamber invasion assay (scale, 100 μm). Mean ± SD, *n* = 5, **p < 0.01, Student’s *t*-test

To identify the specific molecules involved in the prometastatic effect of RPL21, we performed transcriptome sequencing of the RPL21-overexpressing (HCT15, Caco2, HCT116) and RPL21-knockdown (HCT-8, RKO, DLD1) cell lines and their control cells (Fig. 3A). The top 12 differentially expressed genes were selected and their mRNA expression was verified in CRC cells. We found that the mRNA and protein expression of LAMP3 was highly consistent with that of RPL21 (Fig. 3B and Additional file 8: Fig. S3A). Then, we detected the protein expression of LAMP3 in 18 pairs of fresh human CRC tissues (T) and matched adjacent normal tissues (N), and observed higher expression of LAMP3 in tumor tissues, similar to the expression trends for RPL21 (Additional file 8: Fig. S3B). IHC staining of LAMP3 was performed in 142 paraffin-embedded CRC sections. The consecutive slices exhibited apparent consistency in the expression and localization of RPL21 and LAMP3 (Fig. 3C). We also assessed LAMP3 expression in tumor buds and found that it was positively correlated with tumor budding grade (Fig. 3D). Lastly, we analyzed the correlation between the scores of RPL21 and LAMP3 expression in tumor buds and observed a significant positive correlation. (Fig. 3E).

The transwell migration and invasion assays were performed to confirm whether LAMP3 was required to promote the migration and invasion of CRC cells. We observed that silencing of LAMP3 suppressed the migration and invasion of RPL21-overexpressing cells. In contrast, overexpression of LAMP3 restored a part of the migration and invasive ability of RPL21-knockdown cells (Fig. 3F, G). These data suggest the high correlation between LAMP3 and RPL21, and that LAMP3 is essential for promoting the migration and invasion of CRC cells.

### RPL21 upregulates LAMP3 expression by activating TFEB

**Fig. 4:**
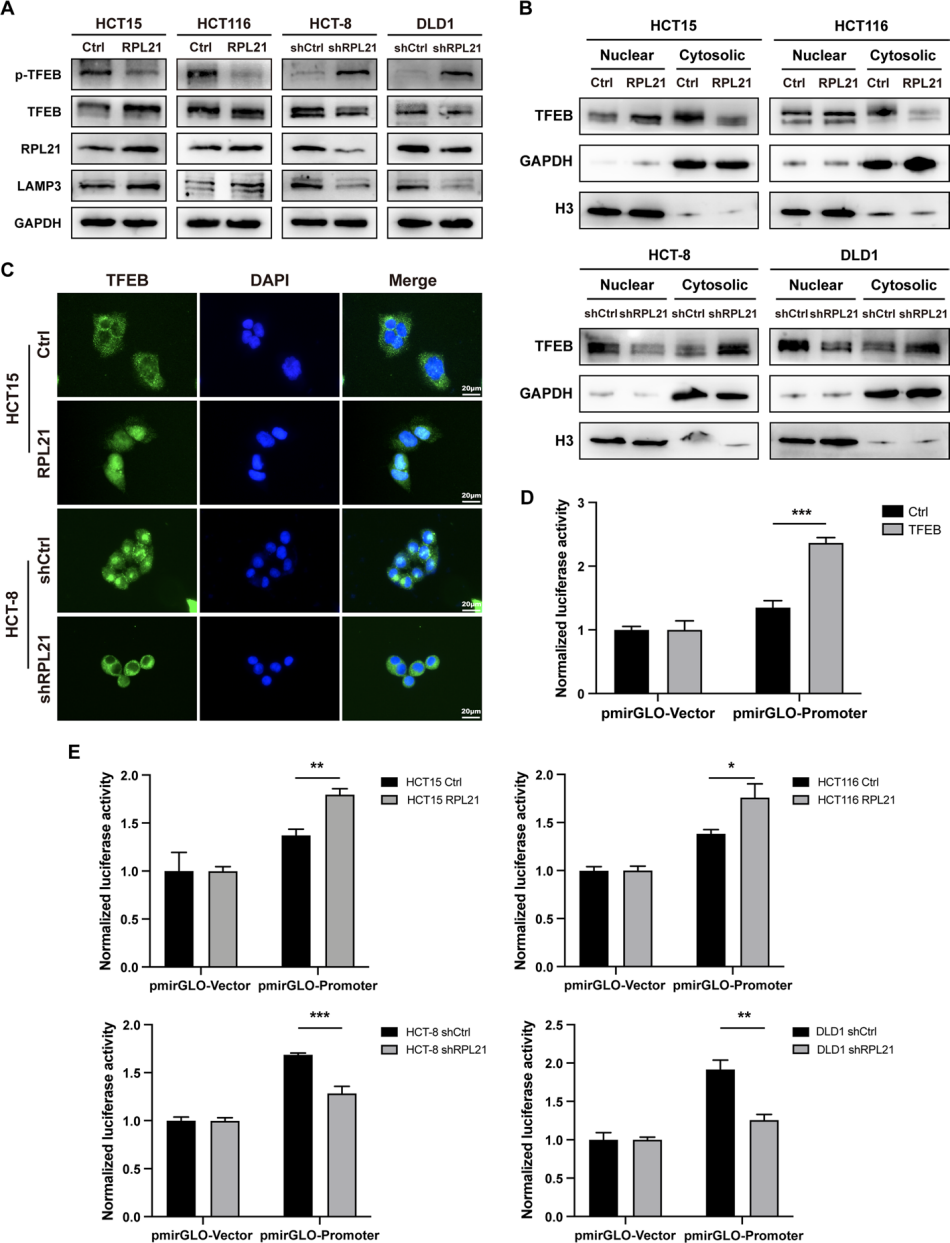
RPL21 upregulates LAMP3 expression by activating TFEB. **A** TFEB, p-TFEB (Ser211), RPL21, and LAMP3 protein expression in the indicated CRC cells was detected by western blotting. **B** The TFEB protein expression in cytosolic and nuclear of the indicated CRC cells was detected by western blotting. The quantification of cytosolic TFEB was normalized to GAPDH, the quantification of nuclear TFEB was normalized to H3. **C** The localization of TFEB (green) in the indicated CRC cells was observed by IF staining (scale, 20 μm). **D** The transcription of *LAMP3* by TFEB was verified by dual-luciferase reporter gene assay in HEK293T cells. Mean ± SD, *n* = 3, ***p < 0.001, Student’s *t*-test. **E** The transcription of *LAMP3* in the indicated CRC cells was verified by dual-luciferase reporter gene assay. Mean ± SD, *n* = 3, ***p < 0.001, **p < 0.01, *p < 0.05, Student’s *t*-test

As we have demonstrated that RPL21 can upregulate LAMP3 expression at the transcriptional level, we explored the specific regulatory mechanism. TFEB is a key transcription factor for lysosomal biogenesis [24]. Its activity is mainly modulated via protein phosphorylation. The phosphorylation of Ser211 can inactivate TFEB and cause cytosolic retention. In contrast, its dephosphorylation results in nuclear translocation and activation of the transcriptional function [25, 26]. We found that compared with the control cells, the expression of pSer211-TFEB decreased in RPL21-overexpressing cells and increased in RPL21-knockdown cells (Fig. 4A). Western blotting and IF staining were performed to investigate the localization of TFEB in CRC cells. We observed apparent cytosolic retention in cells with low RPL21 expression and nuclear localization in cells with high RPL21 expression (Fig. 4B, C). Taken together, these results suggest that RPL21 regulates TFEB activity by inducing dephosphorylation and nuclear translocation.

To further investigate whether TFEB is a crucial factor involved in the upregulation of LAMP3 expression, the dual-luciferase reporter gene assay was performed in HEK293T cells and CRC cells. In HEK293T cells, we observed that TFEB promoted *LAMP3* transcription (Fig. 4D). In CRC cells, we observed that RPL21 contributed to this progress (Fig. 4E). Therefore, RPL21 activates the transcriptional function of TFEB and thereby upregulates LAMP3 expression.

### Direct interaction between RPL21 and LAMP3

**Fig. 5:**
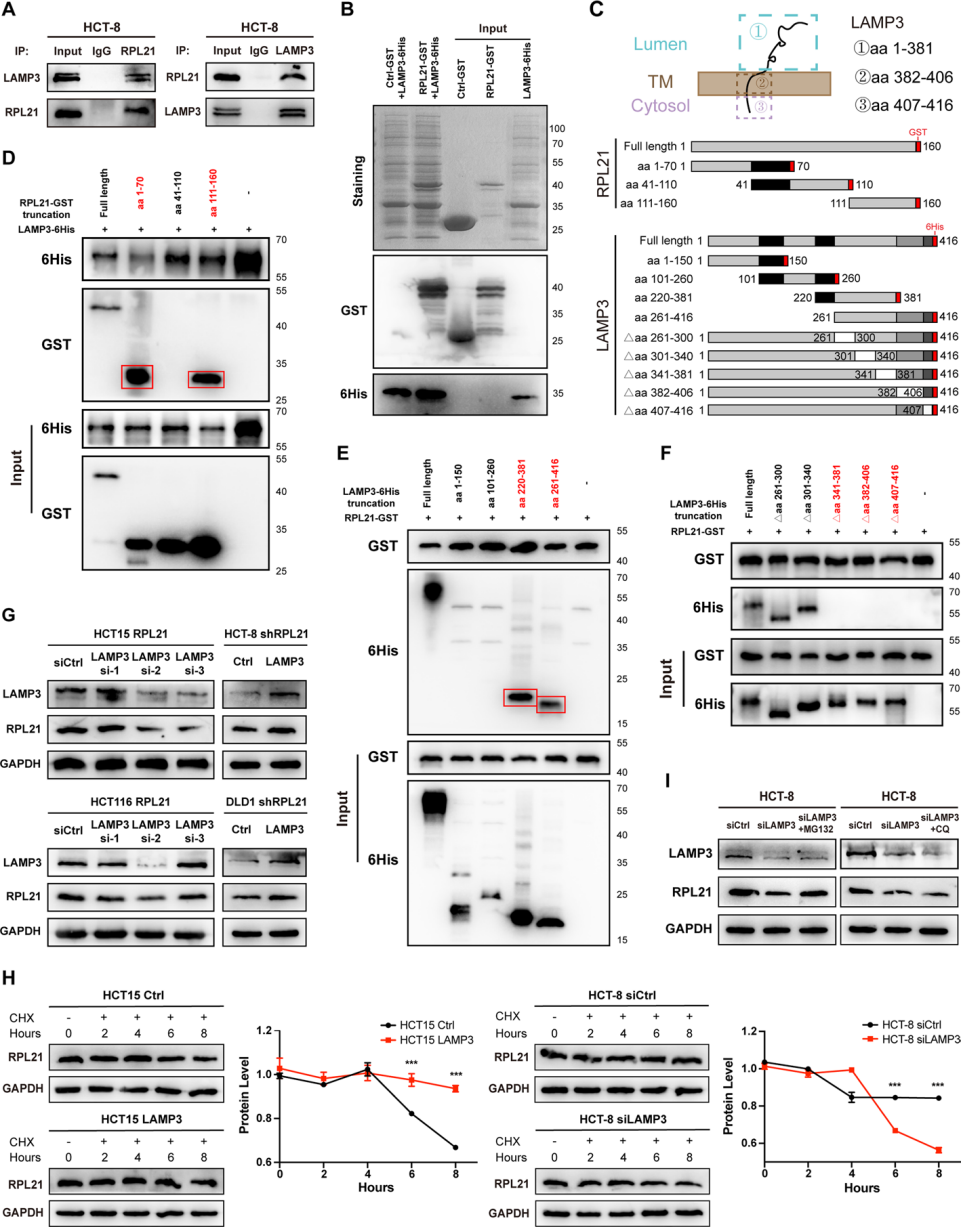
LAMP3 directly interacted with RPL21 and enhanced the stability of the RPL21 protein. **A** RPL21 and LAMP3 were immunoprecipitated in HCT-8 cells detected by western blotting. **B** Direct binding of RPL21-GST and LAMP3-6His using the His pull-down assay. The pull-down bands were detected by Coomassie brilliant blue staining and western blotting. **C** Topology of LAMP3, including the luminal segment (aa 1–381), the transmembrane segment (aa 382–406), and the cytoplasmic segment (aa 407–416). Diagrammatic representation of RPL21 and LAMP3 with their truncated/deleted mutation forms. **D**–**F** HEK293T cells were transfected with the indicated constructs and immunoprecipitated with anti-6His (**D**) or anti-GST (**E**, **F**). The red boxes represent the pull-down bands detected by western blotting. **G** LAMP3 and RPL21 protein expression in the indicated cells was detected by western blotting. **H** The degradation of the RPL21 protein in HCT15 Ctrl, HCT15 LAMP3, HCT-8 siCtrl, and HCT-8 siLAMP3 cells with CHX treatment (50 μg/mL) was detected by western blotting. The quantification of the protein level was normalized to that of GAPDH. Mean ± SD, *n* = 3, ***p < 0.001, Student’s *t*-test. **I** LAMP3 and RPL21 protein expression in HCT-8 siCtrl, HCT-8 siLAMP3, and HCT-8 siLAMP3 cells treated with MG132 (10 μM, 4 h) or CQ (50 μM, 2 h) was detected by western blotting

Co-IP was performed to investigate whether there is an interaction between RPL21 and LAMP3. The interaction between endogenously expressed RPL21 and LAMP3 was confirmed in HCT-8 cells (Fig. 5A). Prokaryotic-expressed RPL21–GST and LAMP3–6His were induced in BL21 cells and purified using GST–tag or His–tag magnetic beads. The direct interaction between RPL21 and LAMP3 was confirmed (Fig. 5B). To precisely localize the binding site, truncations of *RPL21* with GST tag (*RPL21*–GST) and those of *LAMP3* with 6His tag (*LAMP3*–6His) were constructed (Fig. 5C). Three *RPL21*–GST truncations together with *LAMP3*–6His full-length cDNAs were transfected into HEK293T cells and the His pull-down assay was performed. The aa 1–70 and aa 111–160 segments of RPL21 could bind to LAMP3 but the aa 41–110 segment of RPL21 could not (Fig. 5D), indicating that the aa 1–40 and aa 111–160 segments of RPL21 are the binding sites to LAMP3. On the other hand, four *LAMP3*–6His truncations, together with *RPL21*–GST full-length cDNAs, were transfected into HEK293T cells and the GST pull-down assay was performed. The aa 220–381 and aa 261–416 segments of LAMP3 could bind to RPL21 but the aa 1–150 and aa 101–260 segments of LAMP3 could not (Fig. 5E), indicating that aa 261–416 is the binding area of LAMP3 to RPL21. To further identify the specific binding site, five deletion mutations of *LAMP3*–6His were constructed and transfected with *RPL21*–GST full-length cDNAs into HEK293T cells. The GST pull-down assay revealed that the aa 341–416 segment of LAMP3 possibly forms a complete functional domain and is necessary for the direct interaction between RPL21 and LAMP3 (Fig. 5F).

### LAMP3 enhances the stability of the RPL21 protein

During the tightly organized multistep process of ribosome biogenesis, RPs are synthesized in the cytoplasm and imported to the nucleus to assemble into the pre-ribosome with rRNA. These newly synthesized but unassembled RPs are unstable and immediately cleared by the cellular clearance pathways [27]. As RPL21 maintains a high protein level in CRC cells, we hypothesized that there is a protective mechanism that allows the accumulation of the ribosome-free form of RPL21 in the cytoplasm. Therefore, we silenced LAMP3 in RPL21-overexpressing cells and found a decrease in RPL21 protein expression. In contrast, LAMP3 overexpression increased RPL21 protein expression in RPL21-knockdown cells (Fig. 5G). To confirm the protective action of LAMP3 on RPL21 protein degradation, we examined the degradation rate of RPL21. As shown in Fig. 5H, LAMP3 knockdown accelerated the degradation of RPL21 protein, whereas ectopic expression of LAMP3 increased the stability of RPL21 protein. The two main protein degradation pathways identified were the autophagy–lysosome and ubiquitin–proteasome pathways [28]. To identify which pathway is responsible for RPL21 protein degradation and suppressed by LAMP3, LAMP3-knockdown HCT-8 cells were treated with the proteasome inhibitor MG132 or the lysosome inhibitor CQ. We observed that the MG132 treatment could rescue the decrease of RPL21 expression caused by LAMP3 knockdown. In contrast, CQ treatment resulted in no significant change (Fig. 5I). These results indicate that LAMP3 enhances the stability of the RPL21 by suppressing the degradation of the ubiquitin–proteasome system.

### RPL21–LAMP3 regulates FA formation and promotes cell spreading

**Fig. 6:**
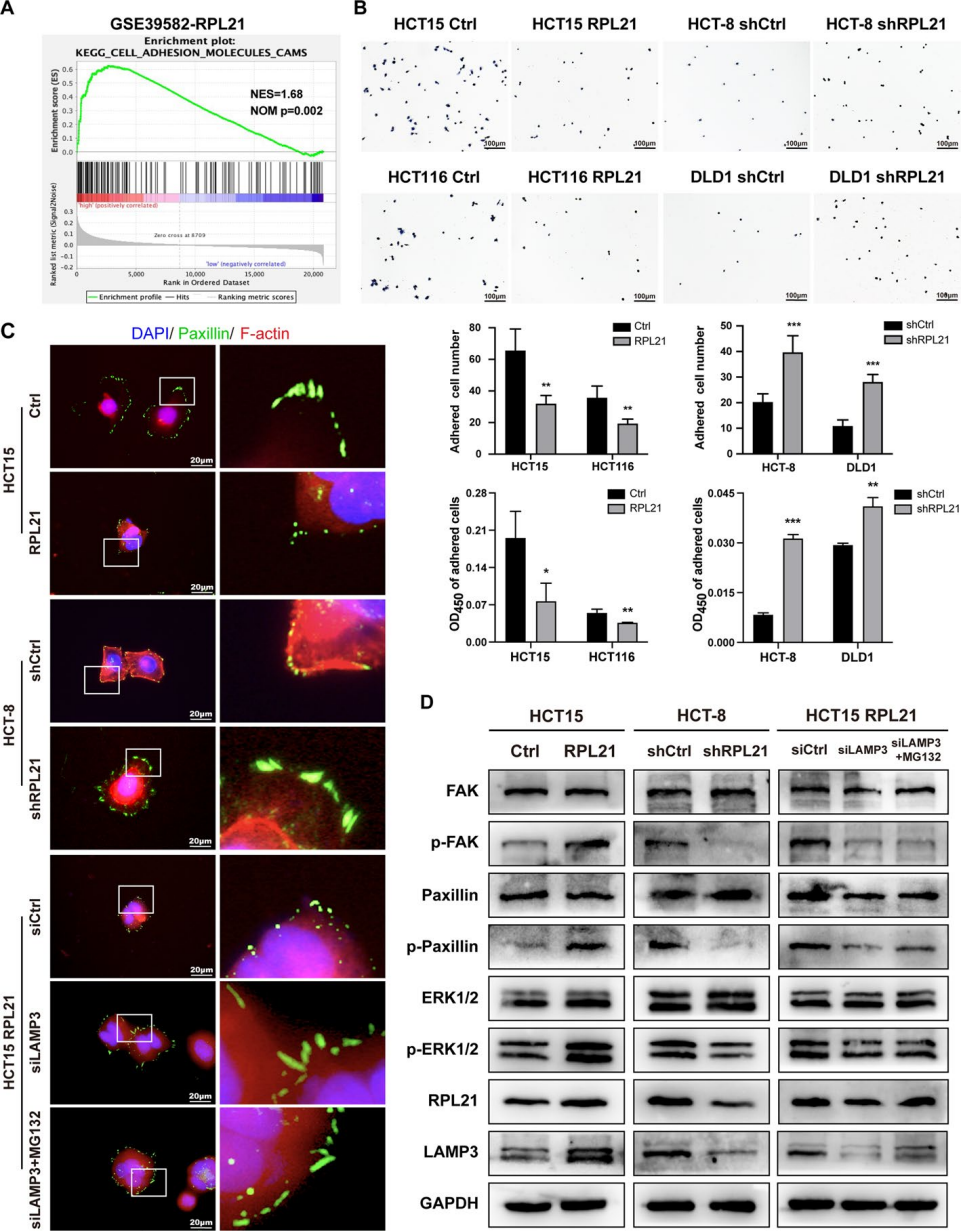
RPL21–LAMP3 regulates FA formation and promotes cell spreading. **A** KEGG enrichment analysis of the RPL21 expression in the CRC database GSE39582. **B** The adhesion ability of the indicated cells was detected by the FN adhesion assay (scale, 100 μm). Mean ± SD, *n* = 5, ***p < 0.001, **p < 0.01, *p < 0.05, Student’s *t*-test. **C** The morphology of FA (paxillin, green) of the indicated cells was observed by IF staining (scale, 20 μm). **D** FAK, pTyr397-FAK, paxillin, pTry118-paxillin, ERK1/2, pThr202/Tyr204-ERK1/2, RPL21, and LAMP3 protein expression in the indicated CRC cells was detected by western blotting

To further identify the molecular mechanism by which RPL21 and LAMP3 promote the migration and invasion of CRC cells, Kyoto Encyclopedia of Genes and Genomes (KEGG) enrichment analysis was performed using the CRC database GSE39582. The adhesion signaling pathway was highly correlated with RPL21 expression (Fig. 6A). The cell adhesion assay confirmed that RPL21 could reduce the adhesion ability of CRC cells (Fig. 6B). Cell adhesion is the process of binding and interacting with neighboring cells or the extracellular matrix via specific molecules. Changes in the cell adhesion ability during cancer progression cause tumor cells to be more dynamic and spread to distant sites, for which the continuous construction and decomposition of FAs are essential [29, 30].

FAs serve as the traction points to transmit strong forces, allowing a net forward translocation of the cell body [31]. Cells with immature FAs, with an unstable structure and few visible adhesion clusters, typically migrate rapidly. In other cells, these small adhesions can mature into larger and more organized adhesions, possibly inhibiting cell migration [32, 33]. Figure 6C shows the IF staining results for paxillin, a major component of FAs. CRC cells with high RPL21 expression had immature FAs, they were small in size, and had nascent adhesions at the leading edge. In contrast, CRC cells with low RPL21 expression displayed mature FAs, which were elongated and tightly adherent. Silencing of LAMP3 led to the conversion of immature FAs to mature FAs. Treatment with MG132 to inhibit RPL21 degradation could not reverse this conversion. These results indicate that RPL21 affects the adhesion ability of CRC cells by regulating FA formation via LAMP3.

To determine whether RPL21 and LAMP3 promote the activation of the FA pathway, western blotting was performed. The results showed that RPL21 could increase pTyr397–FAK, pTyr118–paxillin and pThr202/Tyr204–ERK1/2 in CRC cells. In contrast, LAMP3 inhibition and treatment with MG132 decreased the levels of phosphorylated FAK, paxillin, and ERK1/2 (Fig. 6D), suggesting that RPL21 activates the FA signaling pathway together with LAMP3. These findings demonstrate that RPL21 and LAMP3 promote immature FA formation by activating the FAK/paxillin/ERK signaling pathway, thereby weakening the cell adhesion ability to enhance the migration and invasion of CRC cells.

## Discussion

Cancer cells are known for their ability to continuously grow and their high proliferative activity. The vigorous metabolism of cancer cells requires various proteins, this means they need more active ribosome biosynthesis and more efficient ribosome translational machinery than normal cells [7]. RPs have long been thought to play an essential role in ribosome assembly and protein translation, with little regulatory function. Recent advances in high-throughput analyses have allowed us to identify differentially expressed RP genes in various cancers. This has drawn attention to the functional heterogeneity of individual ribosome-free RPs in tumorigenesis. For example, RPL15 is upregulated in circulating tumor cells in breast cancer. This increases metastatic growth in multiple organs and enhances the translation of other RPs [34]. Further, RPS13 promotes the growth and cell cycle progression of gastric cancer cells by inhibiting p27 expression [35, 36]. These results indicate that RPs can play a tumor-promoting role independent of the translational machinery.

**Fig. 7:**
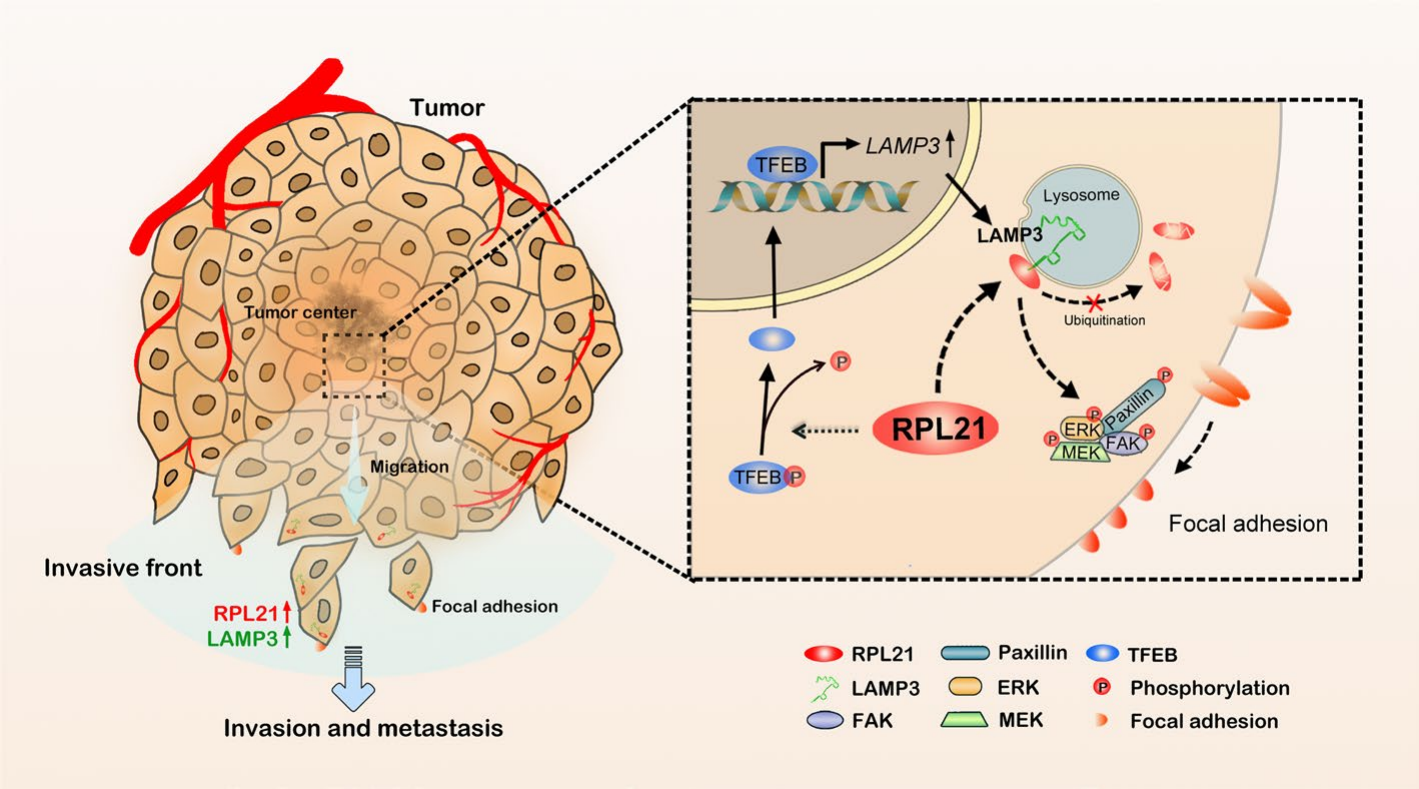
Schematic diagram demonstrating the mechanism of RPL21 and LAMP3 to promote CRC metastasis. The high expression of RPL21 in CRC cells activated the transcriptional function of TFEB to upregulate the expression of LAMP3. The direct interaction between RPL21 and LAMP3 suppressed the ubiquitin–proteasome degradation of RPL21 and activated the FAK/paxillin/ERK signaling pathway to increase the formation of immature FAs, thereby promoting CRC cell migration, invasion, and tumor metastasis

A single-cell transcriptome analysis of 272 CRC epithelial cells and 160 normal epithelial cells led to the identification of 342 discriminative transcripts. RPL21 was the only ribosomal protein transcript among the top 20 most differentially expressed transcripts [37]. High RPL21 expression in CRC was then verified according to public CRC databases. In the present study, we discovered that RPL21 expression in CRC was positively correlated with tumor metastasis and poor patient prognosis. Functional experiments confirmed that RPL21 could promote CRC cell migration and invasion in vitro and tumor metastasis in vivo. Further, high RPL21 expression activated the transcriptional function of TFEB to upregulate LAMP3 expression. The direct interaction between RPL21 and LAMP3 enhanced the protein stability of the RPL21 protein. Further investigation revealed that RPL21 and LAMP3 increased the formation of immature FAs by activating the FAK/paxillin/ERK signaling pathway to promote CRC cell spreading. Taken together, our study results revealed the important role of RPL21, in combination with LAMP3, in CRC metastasis (Fig. 7).

Ribosome biogenesis is the most conserved cellular process, and is subjected to stringent quality control checks. Although RPs are finely balanced and well-coordinated during the physiological processes in normal cells, the reasons for their abnormally high expression in tumors are worth exploring. Different extracellular and intracellular stimulations that disrupt ribosome biogenesis can trigger ribosomal stress (RS), resulting in the massive release of ribosome-free RPs to perform additional functions [38]. These RS-inducing events include but are not limited to chemical agents, radiation, nutrient deprivation, hypoxia, and gene deregulation [39]. In general, as a solid tumor, CRC develops a specific nutrient-deprived and hypoxic microenvironment owing to the rapid growth of tumor cells and the insufficient blood supply [40]. Studies have revealed that in response to abnormal nutrient levels, the mTOR signaling pathway plays a vital role in regulating RPs production [41], and that hypoxia induces the expression of several RPs via alternative mRNA splicing [42]. Whether the accumulation of RPL21 in CRC cells is the consequence of RS caused by nutrient deprivation and/or hypoxia warrants further investigation. The prometastatic effect of RPL21 in CRC, as presented in this study, may be a coping strategy by which CRC cells escape the unfavorable tumor microenvironment.

Specific interaction with some nonribosomal components of the cell, presumably RNA or protein, which has a physiological effect on cells, is one of the criteria for concluding that an RP has extra-ribosomal capacity [43]. In this study, we demonstrated that RPL21 could upregulate LAMP3 expression by activating the transcriptional function of TFEB and directly interacting with it to promote CRC metastasis. LAMP3 is confirmed to be essential for proteasomal degradation and cell survival during proteasome dysfunction [44]. Different factors regulate LAMP3 expression under different conditions. Hypoxia activates the induction of LAMP3 via the PERK/eIF2 alpha arm. Further, proteasome inhibition induces LAMP3 expression in an ATF4-dependent manner in breast cancer cells [19, 45]. Our results demonstrate that mediating TFEB activity is one of the extra-ribosomal functions of RPL21, thereby revealing a new regulatory mechanism of LAMP3 expression in CRC. Further, we observed that combination with LAMP3 benefited RPL21 by suppressing ubiquitin–proteasome degradation to enhance the stability of RPL21 protein. As a result, RPL21 was able to maintain a high protein level to perform other extra-ribosomal functions, including regulation of FA formation. The efficiency of cell motility depends on the stability of FAs [46]. Morphological changes in FAs remodel the adhesive contacts to induce detachment from the extracellular matrix during cell spreading [47]. As we identified the remodeling from large and elongated mature FAs to small and scattered immature FAs and the activation of the FAK/paxillin/ERK signaling pathway in CRC cells, the interaction between RPL21 and LAMP3 was implicated in FA dynamics associated with cell migration. As FA modulation is a considerably complicated biological process, the underlying mechanisms by which RPL21–LAMP3 regulates FA formation remains to be investigated further.

## Conclusions

We found that RPL21 promotes the invasion and metastasis of CRC cells by regulating FA formation in a LAMP3-dependent manner. This study highlights the unique extra-ribosomal function of RPL21 in CRC progression. We have, for the first time, studied the interaction between RPL21 and LAMP3, which are the components of two important organelles, in detail. Our study provides a new perspective to investigate the tumor-promoting mechanism of RPL21 and may help develop novel therapeutic strategies against CRC.

## Supplementary Information

**Additional file 1: Table S1.** The list of primary antibodies used in the study.

**Additional file 2: Table S2.** The list of primer sequences used in the study.

**Additional file 3: Table S3.** The list of RNA interference oligo sequences used in the study.

**Additional file 4: Table S4.** The links to websites and databases used in the study.

**Additional file 5: Figure S1.** The expression of RPL21 in public databases. **A** The RPL21 mRNA expression in different tumor tissues and paired normal tissues from the public database. The red box indicating the expression of RPL21 in COAD. **B**, **C** The analysis of RPL21 mRNA expression in CRC tissues relative to that in normal tissues in CRC GEO databases GSE83889 and GSE87211. Mean ± SD, ***p < 0.001, Student’s *t-*test. **D** The analysis of the correlation between RPL21 mRNA expression and distant metastasis in TCGA-COAD database. Mean ± SD, ***p < 0.001, Student’s *t-*test.

**Additional file 6: Table S5.** Univariate and multivariate Cox proportional hazards models were used to analyze the correlations between individual parameters and CRC relapse-free survival rate in GEO databases GSE39582.

**Additional file 7: Figure S2.** A high expression of RPL21 promotes the migration and invasion of CRC cells. **A** The RPL21 protein expression in 10 CRC cell lines and FHC was detected by Western blotting. The quantification of the protein level was normalized to that of GAPDH. Mean ± SD, *n* = 3, ***p < 0.001, *p < 0.05, Student’s *t-*test. **B** Localization of RPL21 (green) and RPS6 (red) in CRC cells was observed by IF staining (scale, 20 μm). **C** The migration ability of CRC cells was detected by wound healing assay (scale, 200 μm). Mean ± SD, *n* = 5, **p < 0.01, *p < 0.05, Student’s *t-*test. **D** The migration ability of CRC cells was detected by Transwell migration assay (scale, 100 μm). Mean ± SD, *n* = 5, ***p < 0.001, *p < 0.05, Student’s *t-*test. **E** The invasion ability of CRC cells was detected by Matrigel-coated Boyden chamber invasion assay (scale, 100 μm). Mean ± SD, *n* = 5, ***p < 0.001, **p < 0.01, Student’s *t-*test.

**Additional file 8: Figure S3.** LAMP3 was significantly correlated with RPL21. **A** The relative expression of the selected genes mRNA in the indicated CRC cells. Mean ± SD, *n* = 3, **p < 0.01, ***p < 0.001, Student’s *t-*test. **B** The LAMP3 protein expression in human CRC tissues (T) and paired adjacent normal tissues (N) was detected by Western blotting. The quantification of the protein level was normalized to that of GAPDH. *n* = 18, *p < 0.05, paired Student’s *t-*test.

## Data Availability

The datasets used and analyzed during the current study are available from the corresponding authors upon reasonable request.

## Abbreviations

RPL21: Ribosomal protein L21
LAMP3: Lysosome-associated membrane protein 3
CRC: Colorectal cancer
TFEB: Transcription factor EB
FAs: Focal adhesions
RPs: Ribosomal proteins
HHS: Hereditary hypotrichosis simplex
CIN: Cervical intraepithelial neoplasia
siRNA: Small interfering RNA
ATCC: American Type Culture Collection
FBS: Fetal bovine serum
BSA: Bovine serum albumin
IHC: Immunohistochemistry
qRT–PCR: Quantitative reverse transcription polymerase chain reaction
IF: Immunofluorescence
H&E: Hematoxylin–eosin
Co-IP: Co-immunoprecipitation
IPTG: Isopropyl-β-d-1-thiogalactopyranoside
LB: Luria–Bertani
CQ: Chloroquine
KEGG: Kyoto Encyclopedia of Genes and Genomes
FAK: Focal adhesion kinase
ERK: Extracellular signal-regulated kinase
RS: Ribosome stress
