# Supplementary figures and images for: RPL21 interacts with LAMP3 to promote colorectal cancer invasion and metastasis by regulating focal adhesion formation
Source: Cell Mol Biol Lett. 2023 Apr 16;28:31. doi: 10.1186/s11658-023-00443-y (PMC10108486; doi:10.1186/s11658-023-00443-y)

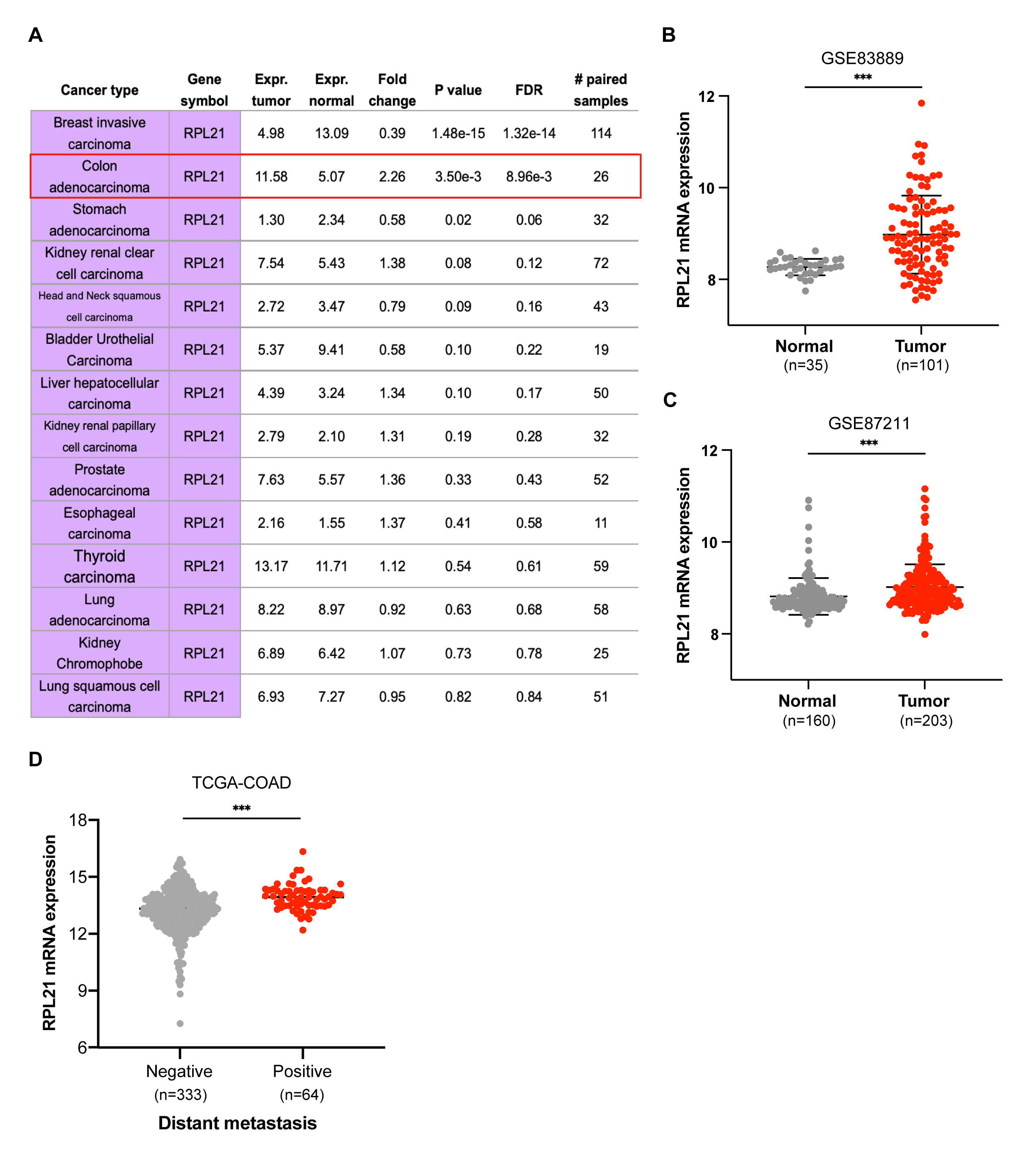

Supplement: Supplementary file 5 — Additional file 5: Figure S1. The expression of RPL21 in public databases. A The RPL21 mRNA expression in different tumor tissues and paired normal tissues from the public database. The red box indicating the expression of RPL21 in COAD. B, C The analysis of RPL21 mRNA expression in CRC tissues relative to that in normal tissues in CRC GEO databases GSE83889 and GSE87211. Mean ± SD, ***p < 0.001, Student’s t-test. D The analysis of the correlation between RPL21 mRNA expression and distant metastasis in TCGA-COAD database. Mean ± SD, ***p < 0.001, Student’s t-test. [file 11658_2023_443_MOESM5_ESM.tif]

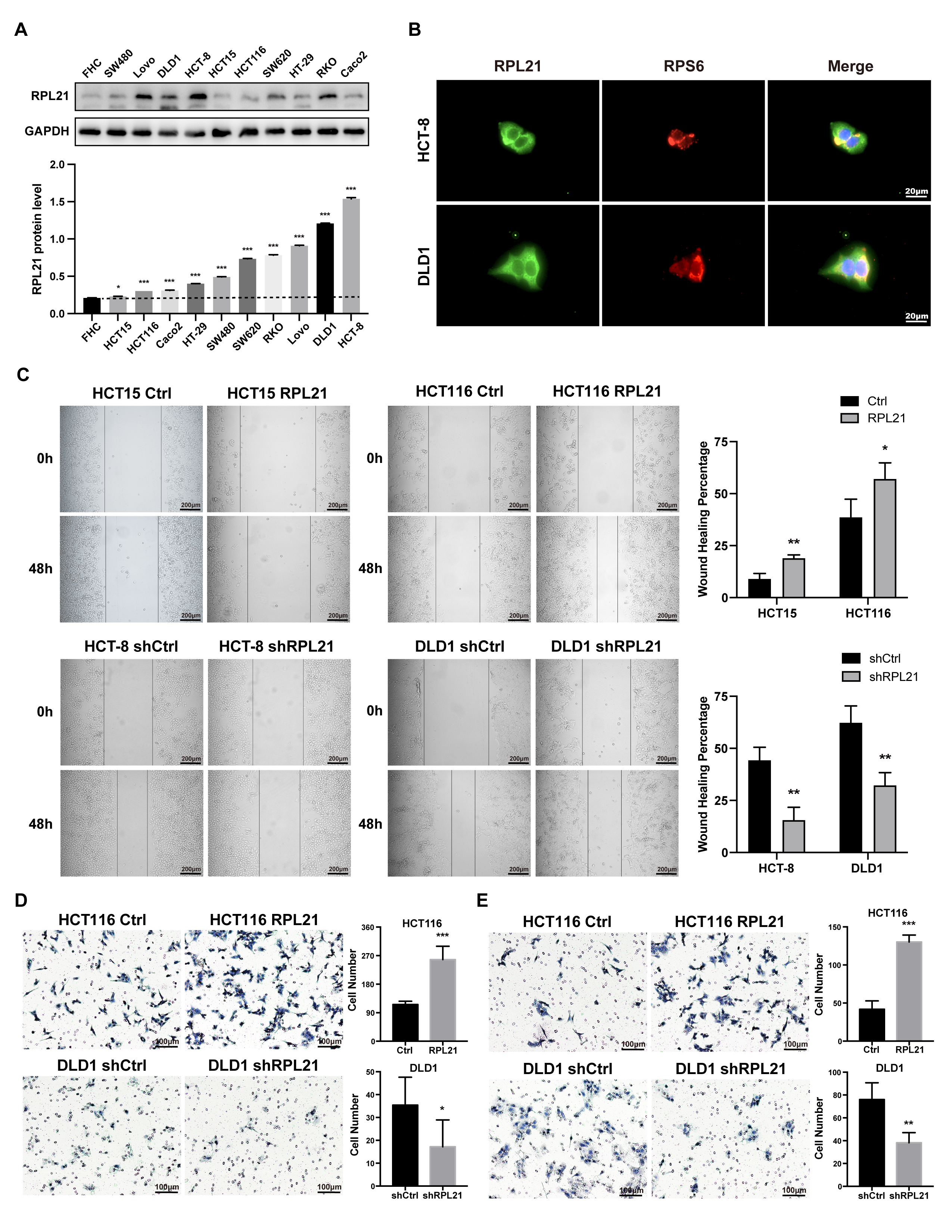

Supplement: Supplementary file 7 — Additional file 7: Figure S2. A high expression of RPL21 promotes the migration and invasion of CRC cells. A The RPL21 protein expression in 10 CRC cell lines and FHC was detected by Western blotting. The quantification of the protein level was normalized to that of GAPDH. Mean ± SD, n = 3, ***p < 0.001, *p < 0.05, Student’s t-test. B Localization of RPL21 (green) and RPS6 (red) in CRC cells was observed by IF staining (scale, 20 μm). C The migration ability of CRC cells was detected by wound healing assay (scale, 200 μm). Mean ± SD, n = 5, **p < 0.01, *p < 0.05, Student’s t-test. D The migration ability of CRC cells was detected by Transwell migration assay (scale, 100 μm). Mean ± SD, n = 5, ***p < 0.001, *p < 0.05, Student’s t-test. E The invasion ability of CRC cells was detected by Matrigel-coated Boyden chamber invasion assay (scale, 100 μm). Mean ± SD, n = 5, ***p < 0.001, **p < 0.01, Student’s t-test. [file 11658_2023_443_MOESM7_ESM.tif]

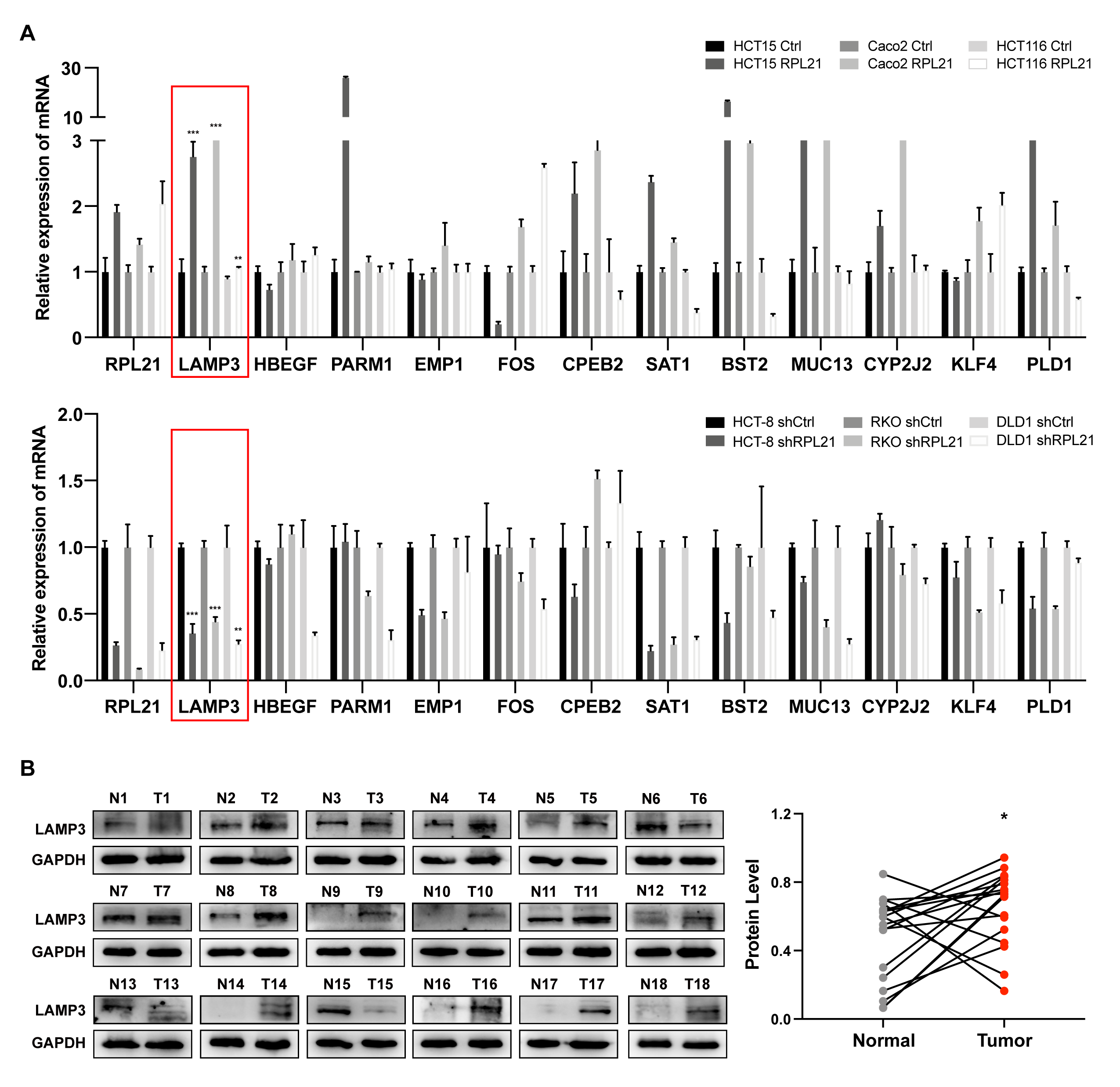

Supplement: Supplementary file 8 — Additional file 8: Figure S3. LAMP3 was significantly correlated with RPL21. A The relative expression of the selected genes mRNA in the indicated CRC cells. Mean ± SD, n = 3, **p < 0.01, ***p < 0.001, Student’s t-test. B The LAMP3 protein expression in human CRC tissues (T) and paired adjacent normal tissues (N) was detected by Western blotting. The quantification of the protein level was normalized to that of GAPDH. n = 18, *p < 0.05, paired Student’s t-test. [file 11658_2023_443_MOESM8_ESM.tif]
